# Progress of Surgery

**Published:** 1900-12-15

**Authors:** 


### Progress of Surgery.

**The Radical Cure of Femoral Hernia.**—T. E. Gordon [5] of Dublin discusses this subject. He points out that it is the upper end of the crural canal which should be occluded, and after trying various devices on the cadaver, he operated on a case in the way described below. The incision began at a point 2 inches from the pubic spine and ¾ inch above Poupart's ligament, and passed at first inwards towards the region of the hernia and then vertically downwards over the hernia. The sac was isolated. Then an opening was made through the external oblique aponeurosis above Poupart's ligament. Through this opening the sac was dislodged from the upper end of the crural canal. The fibres of the internal oblique and transversalis muscles were drawn down by sutures and placed against the pectineus muscle. Also these muscles were fixed to Poupart's ligament by sutures passed at a higher level than the first set, in order to pack down as much muscle fibre as possible against the opening, and to relieve any tension on the first row. The sac was treated somewhat after the manner of Kocher. The opening in the aponeurosis was then closed. The operation seemed very satisfactory, and was easily performed.

**The Radical Cure of Umbilical Hernia.**—Herbert Waterhouse [6] points out that in suturing the linea alba in cases of umbilical hernia it is difficult to obtain good firm union if you merely suture together the fibrous margins of a round hole. He says it is very much better to split longitudinally each half of the linea alba, opening the sheaths of the recti, and then to suture separately, in tiers, (1) the posterior sheaths, (2) the inner margins of the recti, and (3) the anteror sheaths of the same muscles. Operation is advised if there have been attacks of obstruction of the bowels, or when it is known that adherent omentum or bowel is present in the sac. Dr. Piscoli [7] calls attention to a method of radical cure based on the principle of replacing the linea alba by two overlapping layers of the muscular wall. The principle is similar to that used by MacEwen in his operation for inguinal hernia. Piscoli proceeds as follows:—The incision extends two or three inches above and below the hernia. The sac is removed, and its open neck closed with sutures. The peritoneum is separated from the back of the sheaths of the recti with the finger. The linea alba is then cut upwards and downwards for some distance. The hernial opening thus becomes converted into an ellipsoid. The edge of one rectus is then drawn beneath the edge of the other by loop-sutures, like those used in MacEwan's operation, passed about an inch from their margins through the recti and their sheaths. The face edge of the overlapping side is then secured to the sheath of the other by interrupted sutures.

**Strangulated Obturator Hernia.**—J. Munro [8] Elder, of Montreal, reports a successful case of operation for this condition. The rarity of obturator hernia is alluded to, and the statement of Anderson, made in 1896, quoted. Anderson found only one case mentioned in the records of St. Thomas's Hospital from 1870 to 1896, and stated that only thirty cases had been recorded as operated upon in Europe down to 1896. He himself then reported two cases, one of which was bilateral, the recurrence on the second side coming down while the patient was still in hospital under treatment for the original hernia of the opposite side. This patient recovered. R. J. Godlee reported three cases in the *Lancet* for June 1897. He recommended a combined internal and external operation for the affection. Elder's case was that of a spare woman aged seventy-three. Three days before he saw her she had received a sudden jar by missing a step at the bottom of a stairs and had at once felt a severe pain across the lower zone of the abdomen, shooting down the right thigh to the knee. Vomiting soon set in, and by the third day had become fæculent. She had then, in fact, all the symptoms of intestinal obstruction. Laparotomy was performed, and the bowel with some difficulty disengaged from the obturator foramen. The strangulated portion was inky black, but the affected portion extended only three-quarters round the circumference of the bowel and did not involve the mesenteric vessels, i.e., it was a Richter's hernia. No attempt was made to close the foramen. The patient's condition was bad, and the abdominal cavity therefore was filled with hot saline fluid. Elder has found a useful secondary effect of saline fluid, used in this way, in exhausting laparotomies, to be the prevention of the excessive thirst which is apt to torment these patients in the first twenty-four hours after operation. The patient left the hospital, cured, on the twenty-first day after the operation.

**Strangulated Hernia with Volvulus.**—R. Lawford Knaggs [9] reports an interesting example of this association. A woman, aged 61 years, had worn a truss for femoral hernia many years. Herniotomy was performed six days after the rupture had become strangulated. The sac and a knuckle of small intestine were found to be gangrenous. No bowel contents escaped on opening the bowel, and a director could not be passed along the lumen of the bowel beyond the ring. The incision was therefore extended through Poupart's ligament. The loop in the sac was then found to be the chief part of a volvulus, the neck of which lay just within the abdominal cavity. The bowel was resected. Death resulted in twenty-six hours. There was advanced renal disease present. Knaggs remarks that the small loop probably passed into the hernial sac in a reversed position, and the traction exerted on the intra-abdominal ends of the loop led them to become tightly crossed. The loop therefore swelled, and from pressure became gangrenous. This case shows that where an artificial anus has been made, if no relief follows and a probe cannot be passed in the bowel beyond the ring, the incision should be prolonged and the condition of the parts investigated.

[5] Brit. Med. Jour., June 1900. [6] Clin. Jour., June, 1900. [7] Post Grad. Abstract, June, 1900. [8] Annals of Surgery, Aug. 1900. [9] Lancet, June, 1900.

